# Key genes differential expressions and pathway involved in salt and water-deprivation stresses for renal cortex in camel

**DOI:** 10.1186/s12867-019-0129-8

**Published:** 2019-04-08

**Authors:** Yu Cao, Dong Zhang, Huanmin Zhou

**Affiliations:** College of Life Sciences, Inner Mongolia Agricultural University, No. 306 Zhaowuda Road, Hohhot, 010018 China

**Keywords:** *Camelus bactrianu*s, Renal cortex, Salt stress, Water-deprivation stress, Non-coding RNA, Post-transcriptional regulation

## Abstract

**Background:**

Camels possess the characteristics of salt- and drought-resistances, due to the long-time adaption to the living environment in desert. The camel resistance research on transcriptome is rare and deficient, especially reabsorption in renal cortex. Non-coding RNAs are normally considered as the RNA molecules that are not translated into proteins, their current roles remain mostly in regulation of information flux from DNA to protein, further on normal life activities and diseases. In order to reveal the mysterious veil of the post-transcriptional regulation of ncRNAs in renal cortex for the first time as far as we know, we designed and carried out the experiment of salt stress and water-deprivation stress in camel.

**Results:**

By means of RNA-seq in renal cortex of Alxa Bactrian Camel (*Camelus bactrianus*), we identified certain significantly differential RNAs, including 4 novel lncRNAs, 11 miRNAs and 13 mRNAs under salt stress, 0 lncRNAs, 18 miRNAs and 14 mRNAs under water-deprivation stress. By data analysis, the response pathway of post-transcriptional regulation concerning salt and water-deprivation stresses was put forward, involving preventing sodium from entering the cell, purifying of water and compensating neutral amino acids by miR-193b, miR-542-5p interaction with *SLC6A19* mRNA.

**Conclusion:**

Based on the resistance-related lncRNAs, miRNAs, and mRNAs, we proposed the post-transcriptional regulation pathway to explain how camels respond to salt and water-deprivation stresses in the ncRNAs regulation level of renal cortex for the first time, thus hoping to provide a theoretical basis for therapy of disease that is similar to high blood pressure in humans.

**Electronic supplementary material:**

The online version of this article (10.1186/s12867-019-0129-8) contains supplementary material, which is available to authorized users.

## Background

Camel, a kind of animal that living in the desert, has a series of anti-stress characteristics such as the resistance of heat, salt and drought [1–3]. The previous resistance researches of camel have reported that plasma osmolality and renal sodium excretion increased after the saline loading [4], while water-deprivation is correlated with rise in plasma sodium concentration, urine osmolality and sodium excretion, and with concomitant decrease in plasma volume and urine production [5]. Based on the studies in humans and other model species, salt metabolism is regulated by neuroregulation and humoral regulation, especially the two processes of intestinal absorption and renal reabsorption. Either in intestinal epithelial cells or in renal tubules, Na^+^ absorption requires proteins, such as solute carrier family 26 member 3, 6 and 9, EnAC complex and Na^+^/K^+^ ATP enzymes, and Na^+^ absorption is regulated by renin, angiotensin, aldosterone, vasopressin and other hormones in body fluids [6]. In kidney, the formation and excretion of urine are involved in renal cortex and then renal medulla [7]. The solute reabsorption containing Na^+^ reabsorption is involved in the function of renal cortex with high hydraulic conductivity of cortical peritubular capillaries [8, 9]. The water reservation mechanism of renal medulla in camel has also been revealed in our previous study [10]. But the molecular resistance regulation of these crucial features is rarely demonstrated under salt and water-deprivation stress in the renal cortex, especially in the level of non-coding RNA transcriptional regulation. Non-coding RNAs (ncRNAs) are normally considered as the RNA molecules that are not translated into proteins, their current roles remain mostly in regulation of information flux from DNA to protein [11–13]. In summary, we selected long non-coding RNA (lncRNA) and micro RNA (miRNA), the two kinds of ncRNA, for the investigation of regulatory role of mRNA, to explore the camel’s regulation in response to the environmental stress of salt and water-deprivation. Furthermore, concerning that the resistance-related genes and pathway may be interrelated between camel and human, we hope to provide inspirations of diagnostic and therapeutic strategies for human diseases such as high blood pressure from scientific experimental evidences.

## Results

### Differently expressed protein-coding genes under salt and water-deprivation stresses

**Fig. 1:**
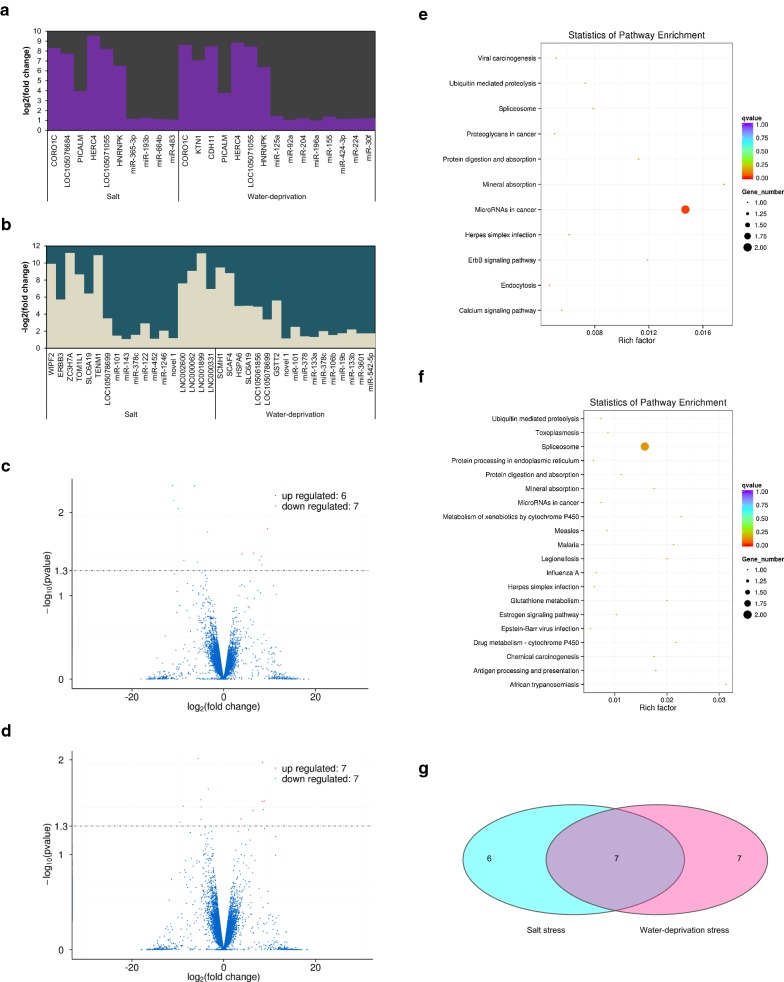
Differentially expressed mRNAs, miRNAs, lncRNAs and the analysis of protein-coding genes within KEGG pathways. **a** Values of log2(fold change) of up-regulated mRNAs and miRNAs under salt and water-deprivation stresses. **b** Values of − log2(fold change) of down-regulated mRNAs, miRNAs and lncRNAs under salt and water-deprivation stresses. **c** Number of differentially expressed protein-coding genes under salt stress. **d** Number of differentially expressed protein-coding genes under water-deprivation stress. **e** Enriched KEGG pathways of protein-coding genes in the renal cortex under salt stress. **f** Enriched KEGG pathways of protein-coding genes in the renal cortex under water-deprivation stress. **g** Seven overlapped protein-coding genes of differential expression under salt and water-deprivation stresses

Salt resistance-related genes with significantly different expression were involved in 13 genes, including up-regulated *CORO1C*, *LOC105076684*, *PICALM*, *HERC4*, *LOC105071055*, *HNRNPK* and down-regulated *WIPF2*, *ERBB3*, *ZC3H7A*, *TOM1L1*, *SLC6A19*, *TENM1*, *LOC105078699* through RNA-seq analysis of camel renal cortex (Fig. 1a–c; Additional file 1: Table S1). Under water-deprivation stress, there were 14 differently expressed genes which referred to up-regulated *CORO1C*, *KTN1*, *CDH11*, *PICALM*, *HERC4*, *LOC105071055*, *HNRNPK* and down-regulated *SCMH1*, *SCAF4*, *HSPA6*, *SLC6A19*, *LOC105061856*, *LOC105078699*, *GSTT2* in renal cortex of camel (Fig. 1a, b, d; Additional file 1: Table S2). Any pathways associated with sodium/water-metabolism were not sorted out in Kyoto Encyclopedia of Genes and Genomes (KEGG) Pathway enrichment analysis, except Mineral absorption with *SLC6A19* (Fig. 1e, f). Moreover, *SLC6A19* was also discovered in 7 overlapped genes under salt and water-deprivation stresses using Venn diagram (Fig. 1g).

### miRNAs identification and candidate interaction with miRNA–mRNA

**Fig. 2:**
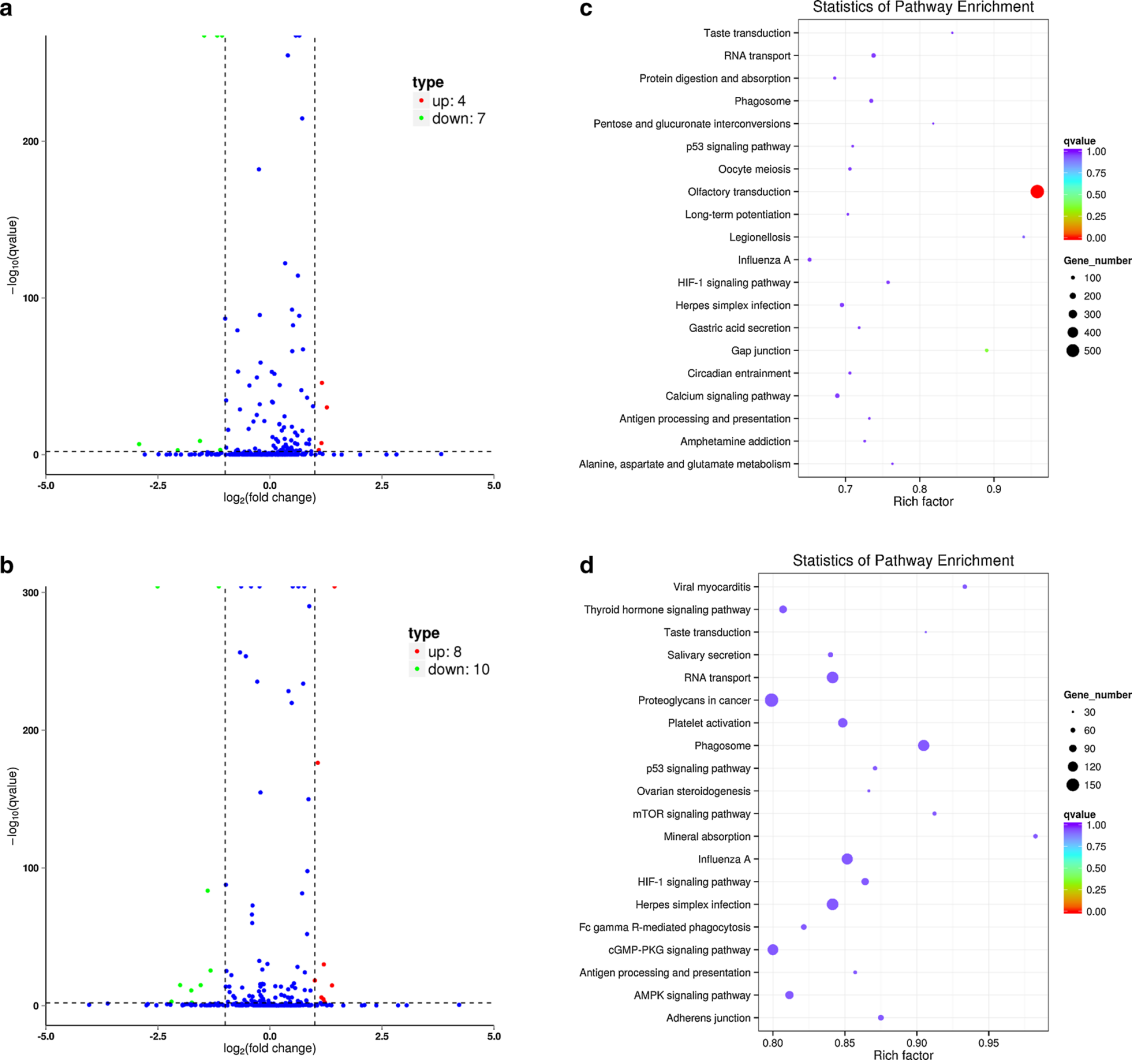
Number of differentially expressed miRNAs and KEGG pathways of target mRNAs. **a** Number of differentially expressed miRNAs under salt stress. **b** Number of differentially expressed miRNAs under water-deprivation stress. **c** Enriched KEGG pathways of target mRNAs in the renal cortex under salt stress. **d** Enriched KEGG pathways of target mRNAs in the renal cortex under water-deprivation stress

The significantly differential expression of miRNAs under salt and water-deprivation stresses was obtained by RNA-seq and sequence alignment with *Bos Taurus*’s miRNA (Fig. 2a, b; Additional file 1: Tables S1, S2). Four significantly up-regulated miRNAs (miR-365-3p, miR-193b, miR-664b and miR-483) and seven significantly down-regulated miRNAs (miR-101, miR-143, miR-378c, miR-122, miR-452, miR-1246 and novel 1) were detected in the camel renal cortex under salt stress (Fig. 1a, b). Eight significantly up-regulated miRNAs (miR-125a, miR-92a, miR-204, miR-196a, miR-155, miR-424-3p, miR-224 and miR-30f) and ten significantly down-regulated miRNAs (miR-101, miR-378, miR-133a, miR-378c, miR-106b, miR-19b, miR-133b, miR-3601, miR-542-5p and novel 1) were discovered in the camel renal cortex under water-deprivation stress (Fig. 1a, b). Mineral absorption was enriched by KEGG pathway analysis of target mRNAs in terms of sodium/water-metabolism (Fig. 2c, d). Nevertheless, *SLC6A19* mRNA was a corresponding target of miR-193b and miR-542-5p by miRNA target prediction using miRanda.

### Novel lncRNAs under salt stress and water-deprivation stress

**Fig. 3:**
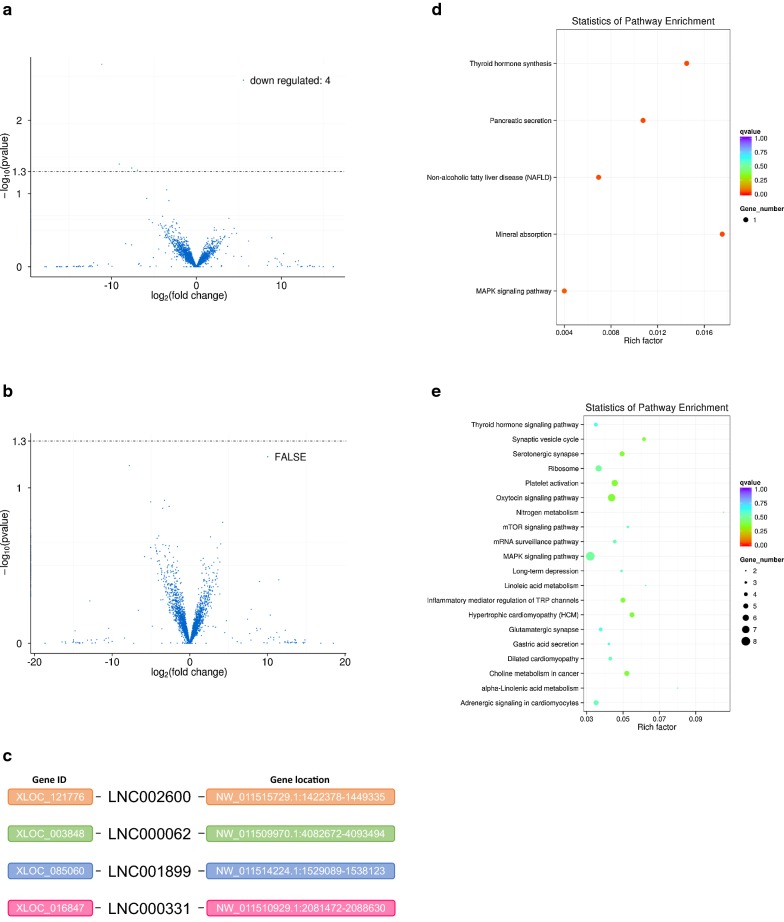
Four novel lncRNAs and KEGG pathways of co-located and co-expressed mRNAs. **a** Number of differentially expressed lncRNAs under salt stress. **b** Number of differentially expressed lncRNAs under water-deprivation stress. **c** Detail genes information of differentially expressed lncRNAs. **d**, **e** Enriched KEGG pathways of co-located and co-expressed mRNAs in the renal cortex under salt stress

For camel renal cortex under salt stress and water-deprivation stress, the differentially expressed lncRNA was presented by RNA-seq (Fig. 3a, b; Additional file 1: Tables S1, S2). The data of RNA-seq indicated that four novel significantly down-regulated lncRNAs (LNC002600, LNC000062, LNC001899 and LNC000331) were detected under salt stress, utilizing sequence alignment with *Bos Taurus*’s lncRNA (Figs. 1a, 3c; Additional file 1: Table S3). Whereas, there was no significant difference in the expression of lncRNAs under water-deprivation stress (Fig. 3b). The co-located and co-expressed mRNAs were predicted, and KEGG pathway analysis of the mRNAs was carried out accordingly with the novel lncRNAs by bioinformatics analysis (Fig. 3d, e). There are two explanations for the RNA-seq results that differentially expressed lncRNAs were not detected under water-deprivation stress. Firstly, the renal cortex may not be a major regulation section of water-deprivation. Secondly, we did not sequence circular RNAs (circRNAs) which may be differentially expressed and execute essential bio-function, not lncRNAs.

## Discussion

**Fig. 4:**
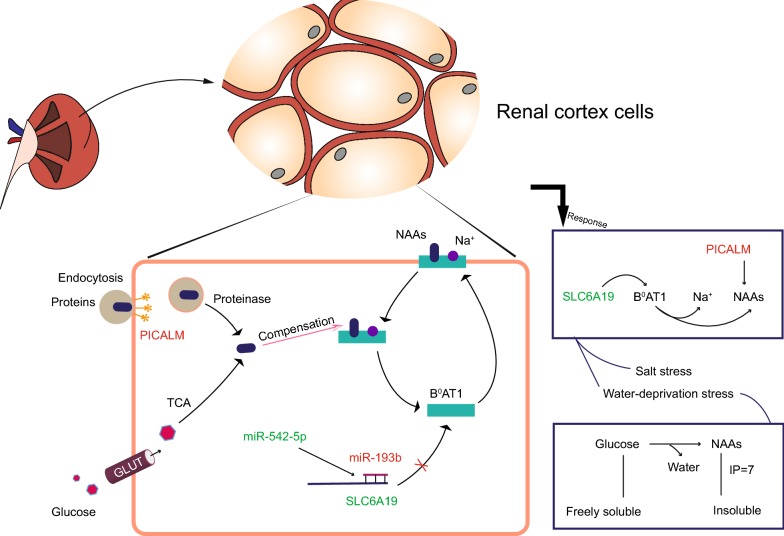
The post-transcriptional regulation pathway to positively respond to salt and water-deprivation stresses in the renal cortex of camel

In order to explore the corporate regulation of camel to deal with the salt and water-deprivation stresses, we found a directly related mRNA with sodium transport in the common 7 protein-coding genes, namely *SLC6A19*. For the *SLC6A19*, we conducted a combined analysis of lncRNA–miRNA–mRNA in the post-transcriptional regulation level by the competing endogenous RNA model [14, 15]. However, we did not discover the co-located and co-expressed lncRNAs with *SLC6A19*. But there were two miRNAs, up-regulated miR-193b under salt stress and down-regulated miR-542-5p under water-deprivation stress, targeting *SLC6A19* mRNA by target gene prediction. In miRNA–mRNA interaction model [16], miR-193b will bind to 3′ untranslated region (3′ UTR) of *SLC6A19* mRNA to inhibit the translation of *SLC6A19*. According to the miRNA-mediated mechanism of extending mRNA half-life [17, 18], the down-regulated expression of miR-542-5p will fail to prevent *SLC6A19* mRNA from degradation. Based on previous studies, neutral amino acids (NAAs) are transported by broad neutral (^0^) amino acid transporter 1 (B^0^AT1), encoded by *SLC6A19*, which is a kind of Na^+^-dependent transporters in the kidney [19, 20]. Glucose transporters (GLUTs) are a large group of membrane proteins. Their essential function is to accelerate the transport of glucose through a plasma membrane [21, 22]. By bioinformatics analysis of screened resistance-related genes, the post-transcriptional regulation pathway under salt and water-deprivation stresses in renal cortex of camel is illustrated (Fig. 4): the inhibition of *SLC6A19* mRNA by up-regulated miR-193b and down-regulated miR-542-5p prevents excessive sodium ions from entering the renal cortical cells, and compensates for NAAs by tricarboxylic acid cycling of glucose which enters the cells according to the concentration gradient. *PICALM* which was also detected in the common 7 mRNAs, can encode phosphatidylinositol-binding clathrin assembly protein and participate in clathrin-mediated endocytosis [23, 24]. The endocytosis of NAAs-contained proteins also assists in compensating NAAs via proteinase.

The above regulation pathway can be able to reduce excessive sodium ions into the cells of renal cortex, and reduce the amount of glucose which is well soluble in water to improve the amount of intracellular NAAs and purify water since the isoelectric point of NAAs is close to 7 (low solubility in water) [25]. The absorption of NAAs in camel renal cortex may not directly transport free NAAs into the cell through B^0^AT1, but count on direct endocytosis of the protein and tricarboxylic acid (TCA) cycle of glucose to achieve the purpose of compensating neutral amino acids.

## Methods

### Stress treatment of camel

Nine Alxa Bactrian Camels were randomly divided into three groups of salt stress (experimental group), water-deprivation stress (experimental group), and free diet (control group), and treated for a period of 24 days  (Additional file 2: Figure S1). Each group included three camels. Salt stress group was treated as follows: salt intake base was 200 g/day, and increased by 100 g every 3 days. Consequently, the formula of salt intake corresponding to salt stress days is as follows: a_n_ = 200 g + 100 g * int[(n − 1)/3], a_n_ refers to the amount of feeding salt, n means salt stress days [26, 27]. Water-deprivation group was free feed intake but fasting water.

### Sample collection and preparation

We applied mercy-killing to the nine Alxa Bactrian Camels of three groups by carotid artery letting blood after intramuscular injection of 0.5 mg/kg xylazine [28], then collected renal cortex tissues separately. The renal cortex samples were stored in 1.5 mL frozen tubes and deposited in liquid nitrogen (− 196 °C) for preservation. The samples were sent to Novogene Corporation (Tianjin, China) by freezing in the dry ice for the next procedure.

### RNA isolation and quantification

Total RNA was extracted from renal cortex tissues of the nine Alxa Bactrian Camels using RNeasy Mini Kit (QIAGEN, Germany). RNA degradation and contamination were monitored on 1% agarose gels. RNA purity was checked using the NanoPhotometer spectrophotometer (IMPLEN, CA, USA). RNA concentration was measured using Qubit RNA Assay Kit in Qubit 2.0 Fluorometer (Life Technologies, CA, USA). RNA integrity was assessed using the RNA Nano 6000 Assay Kit with the Agilent Bioanalyzer 2100 system (Agilent Technologies, CA, USA).

### Library preparation for small RNA and lncRNA sequencing

A total amount of 3 μg total RNA per sample was used separately as input material for the small RNA and lncRNA library. Sequencing libraries of small RNA were generated using NEBNext Multiplex Small RNA Library Prep Set for Illumina (NEB, USA.). For lncRNA library preparation, firstly, ribosomal RNA was removed by Epicentre Ribo-zero rRNA Removal Kit (Epicentre, USA), and rRNA free residue was cleaned up by ethanol precipitation. Subsequently, sequencing libraries of lncRNA were generated using the rRNA-depleted RNA by NEBNext Ultra Directional RNA Library Prep Kit for Illumina (NEB, USA).

### Clustering and sequencing for small RNA and lncRNA

The clustering of the index-coded samples was performed on a cBot Cluster Generation System using TruSeq SR Cluster Kit v3-cBot-HS (Illumia) for small RNA and TruSeq PE Cluster Kit v3-cBot-HS (Illumia) for lncRNA. By pooled RNA-seq [29], the library constructions of small RNA and lncRNA were performed on an Illumina HiSeq 2500 platform and Illumina HiSeq 4000 platform, respectively.

### Data analysis for miRNA and lncRNA

The analyses procedures of miRNA data were as follows: quality control, reads mapping to *Camelus Bactrianus* genome by Bowtie [30], novel miRNA prediction by miREvo [31] and mirdeep2 [32], differential expression analysis by TPM [33] and DEGseq [34], small RNA annotation by *Bos Taurus* database, target gene prediction by miRanda, GO and KEGG pathway enrichment analyses of target genes by GOseq [35] and KOBAS (2.0) [36]. The analyses procedures of lncRNA data were as follows: quality control, reads mapping to *C. Bactrianus* genome by TopHat (v2.0.9) [37], transcript assembly by Cufflinks [38], lncRNA and mRNA sorting by CNCI (v2) [39], CPC (0.9-r2) [40], Pfam Scan (v1.3) [41, 42], PhyloCSF (v20121028) [43], mRNA and lncRNA annotation by *C. Bactrianus* and *Bos Taurus* databases, differential expression analysis by Cuffdiff (http://cole-trapnell-lab.github.io/cufflinks/cuffdiff/index.html), target gene prediction by lncRNA gene upstream/downstream 100 kb and Pearson Correlation Coefficient [44], GO and KEGG pathway enrichment analyses of target genes and differentially expressed mRNAs by GOseq [35] and KOBAS (2.0) [36].

## Conclusion

Based on the lncRNA, miRNA, and mRNA data by RNA-seq and incorporated previous studies, we identified four novel salt-resistance-related lncRNAs in renal cortex and proposed the ncRNAs-related post-transcriptional regulation pathway to explain how camels respond to salt stress and water-deprivation stress for the first time, in light of differentially expressed *SLC6A19*, miR-193b and miR-542-5p. We are hoping to provide a theoretical basis for healing disease that is similar to high blood pressure for humans. Due to the defects in the camel genome function, we selected the database of the bovine which is close to the camel for the annotations of lncRNA and miRNA in this experiment, so we still need more evidences to optimize the results in the future .

## Additional files

**Additional file 1: Table S1.** The differentially expressed mRNAs, miRNAs and lncRNAs of renal cortex under salt stress. **Table S2.** The differentially expressed mRNAs and miRNAs of renal cortex under water-deprivation stress. **Table S3.** Sequence of significantly down-regulated four novel lncRNAs in the renal cortex of camel under salt stress.

**Additional file 2: Figure S1.** Normalized mean plasma Na^+^ concentration of camel under salt stress, water-deprivation stress and free diet.

## Abbreviations

B^0^AT1: broad neutral (^0^) amino acid transporter 1
ceRNAs: competing endogenous RNAs
circRNAs: circular RNAs
GLUTs: glucose transporters
KEGG: Kyoto Encyclopedia of Genes and Genomes
lncRNA: long non-coding RNA
miRNA: micro RNA
NAAs: neutral amino acids
ncRNAs: non-coding RNAs
TCA: tricarboxylic acid
3′ UTR: 3′ untranslated region
