# Supplementary material for: Key genes differential expressions and pathway involved in salt and water-deprivation stresses for renal cortex in camel
Source: BMC Mol Biol. 2019 Apr 8;20:11. doi: 10.1186/s12867-019-0129-8 (PMC6454748; doi:10.1186/s12867-019-0129-8)
Supplement: Supplementary file 1 — Additional file 1: Table S1. The differentially expressed mRNAs, miRNAs and lncRNAs of renal cortex under salt stress. Table S2. The differentially expressed mRNAs and miRNAs of renal cortex under water-deprivation stress. Table S3. Sequence of significantly down-regulated four novel lncRNAs in the renal cortex of camel under salt stress. [file 12867_2019_129_MOESM1_ESM.docx]

**Table S1**. The differentially expressed mRNAs, miRNAs and lncRNAs of renal cortex under salt stress

| **Name** | **SSRC FPKM** | **CRC FPKM** | **log2(foldchange)** | **pvalue** | **qvalue** |
| --- | --- | --- | --- | --- | --- |
| CORO1C | 5.85528 | 0.0184558 | 8.30952 | 0.0425719 | 1 |
| LOC105076684 | 1.41189 | 0.00664148 | 7.73191 | 0.0372037 | 1 |
| PICALM | 18.7315 | 1.19868 | 3.96595 | 0.0315375 | 0.967167 |
| HERC4 | 6.0042 | 0.00805006 | 9.54275 | 0.0157431 | 0.716139 |
| LOC105071055 | 4.98314 | 0.0167936 | 8.213 | 0.033594 | 0.991246 |
| HNRNPK | 30.4609 | 0.334004 | 6.51095 | 0.0307323 | 0.957199 |
| WIPF2 | 0.00454167 | 4.35096 | -9.9039 | 0.00905785 | 0.550872 |
| ERBB3 | 0.189701 | 10.005 | -5.72085 | 0.039552 | 1 |
| ZC3H7A | 0.00530508 | 12.3092 | -11.1801 | 0.00479342 | 0.402007 |
| TOM1L1 | 0.0170656 | 6.92052 | -8.66365 | 0.0377073 | 1 |
| SLC6A19 | 0.958701 | 81.9985 | -6.41837 | 0.00482591 | 0.40333 |
| TENM1 | 0.00139122 | 2.68742 | -10.9157 | 0.00720346 | 0.493081 |
| LOC105078699 | 149.443 | 1692.84 | -3.50179 | 0.0172645 | 0.747318 |
| miR-101 | 6299.10687 | 17416.98709 | -1.4673 | 0 | 0 |
| miR-143 | 20465.7353 | 42909.4866 | -1.0681 | 0 | 0 |
| novel 1 | 5543.493695 | 12534.1481 | -1.177 | 0 | 0 |
| miR-365-3p | 862.4393158 | 386.2562877 | 1.1589 | 9.89E-48 | 1.46E-46 |
| miR-193b | 494.280639 | 204.8307815 | 1.2709 | 6.67E-32 | 7.11E-31 |
| miR-378c | 43.48551025 | 128.3827819 | -1.5618 | 2.82E-10 | 1.87E-09 |
| miR-664b | 139.684967 | 63.01420254 | 1.1484 | 6.60E-09 | 4.23E-08 |
| miR-122 | 5.732816463 | 43.34823164 | -2.9187 | 3.06E-08 | 1.83E-07 |
| miR-452 | 36.63409545 | 79.07936187 | -1.1101 | 0.000192 | 0.00094524 |
| miR-483 | 60.68395964 | 28.52950709 | 1.0889 | 0.00023827 | 0.0011437 |
| miR-1246 | 6.711590006 | 27.97553607 | -2.0594 | 0.00031301 | 0.0014481 |
| LNC002600 | 0.00263165 | 0.514832 | -7.61199 | 0.0448036 | 1 |
| LNC000062 | 0.0015827 | 0.854116 | -9.0759 | 0.0396646 | 1 |
| LNC001899 | 0.118063 | 266.947 | -11.1428 | 0.00172831 | 0.234243 |
| LNC000331 | 0.0219575 | 2.71202 | -6.94851 | 0.047946 | 1 |

SSRC and CRC indicate salt stress renal cortex and control renal cortex, respectively.

**Table S2**. The differentially expressed mRNAs and miRNAs of renal cortex under water-deprivation stress

| **Name** | **WDSRC FPKM** | **CRC FPKM** | **log2(foldchange)** | **pvalue** | **qvalue** |
| --- | --- | --- | --- | --- | --- |
| CORO1C | 7.29255 | 0.0184558 | 8.62621 | 0.0335223 | 0.990625 |
| KTN1 | 0.880792 | 0.0065968 | 7.06089 | 0.0497106 | 1 |
| CDH11 | 2.7916 | 0.00789668 | 8.46563 | 0.0106521 | 0.596393 |
| PICALM | 16.3088 | 1.19868 | 3.76614 | 0.0419573 | 1 |
| HERC4 | 3.7333 | 0.00805006 | 8.85724 | 0.0272385 | 0.909719 |
| LOC105071055 | 5.87575 | 0.0167936 | 8.45072 | 0.02744 | 0.912984 |
| HNRNPK | 27.8888 | 0.334004 | 6.38368 | 0.0342225 | 0.998588 |
| SCMH1 | 0.00532612 | 3.77453 | -9.469 | 0.0454806 | 1 |
| SCAF4 | 0.0109941 | 4.93565 | -8.81037 | 0.0308638 | 0.95902 |
| HSPA6 | 0.21369 | 6.61036 | -4.95114 | 0.0423391 | 1 |
| SLC6A19 | 2.60773 | 81.9985 | -4.97473 | 0.0264411 | 0.898279 |
| LOC105061856 | 1.62051 | 47.5858 | -4.87601 | 0.0314148 | 0.965768 |
| LOC105078699 | 163.481 | 1692.84 | -3.37226 | 0.020395 | 0.804311 |
| GSTT2 | 2.7787 | 134.77 | -5.59994 | 0.00969819 | 0.570224 |
| miR-101 | 2795.805269 | 15922.66129 | -2.5097 | 0 | 0 |
| miR-125a | 9483.696841 | 3490.777701 | 1.4419 | 0 | 0 |
| novel 1 | 5189.492136 | 11458.7554 | -1.1428 | 0 | 0 |
| miR-92a | 6247.594881 | 2982.436601 | 1.0668 | 3.08E-178 | 5.13E-177 |
| miR-378 | 395.1649681 | 1035.673773 | -1.39 | 3.39E-85 | 4.00E-84 |
| miR-204 | 832.1817373 | 361.4729367 | 1.203 | 1.38E-31 | 1.22E-30 |
| miR-133a | 133.2960185 | 334.2516823 | -1.3263 | 4.34E-27 | 3.51E-26 |
| miR-196a | 734.5712671 | 367.0437981 | 1.001 | 7.68E-20 | 5.57E-19 |
| miR-378c | 29.25690169 | 117.3679202 | -2.0042 | 1.95E-16 | 1.26E-15 |
| miR-106b | 54.70909419 | 159.6558225 | -1.5451 | 2.22E-16 | 1.40E-15 |
| miR-155 | 309.493413 | 118.3808041 | 1.3865 | 3.58E-16 | 2.20E-15 |
| miR-19b | 29.78168916 | 100.5287257 | -1.7551 | 1.87E-12 | 9.14E-12 |
| miR-424-3p | 176.5909851 | 79.76460599 | 1.1466 | 2.80E-07 | 1.08E-06 |
| miR-224 | 125.6866001 | 54.9489508 | 1.1937 | 6.52E-06 | 2.34E-05 |
| miR-133b | 4.329496663 | 19.87784626 | -2.1989 | 0.00036606 | 0.0011515 |
| miR-30f | 63.49928439 | 27.22125443 | 1.222 | 0.0010429 | 0.0031411 |
| miR-3601 | 5.641465348 | 18.86496237 | -1.7416 | 0.002389 | 0.0069015 |
| miR-542-5p | 5.641465348 | 18.86496237 | -1.7416 | 0.002389 | 0.0069015 |

WDSRC and CRC indicate water-deprivation stress renal cortex and control renal cortex, respectively.

**Table S3**. Sequence of significantly down-regulated four novel lncRNAs in the renal cortex of camel under salt stress.

|  | Sequence |
| --- | --- |
| LNC 002600 | GATTGCTTCTGTCATATCGCTTTGCTGTTTTGGAGTGTACTTAAAGGATTATGATTGATAAGGCTGTCTTGCCAGAGAATGTTGACACAGaatCTGCAGTTCATAATGGAGCTACTGTGCTGGCTTTGGAAGGAGGCGATTCTGAAGATAGAGAGattcagCTGTCGGTCTTTGACATTTCCTGTGGTCTGTTCACTCTAGATGCAAAGGACATGGAAAAATCAGgagcCTTCTTATTCAAAACACCTTTCAAACTCAAGGCATAAAAATAGCAGGCAGCTTCCACTTGCAGGCTTTTGAAGCTGGAGAAGACTTGTTTTTCATGTGAAGAAACTGCATCCCAGGAAGATGAGATGACTTGCTAGTAAGTGATAATCAGGATCTTTTGACTCCAGAGCAAACTCTGCCAACTTGAGGATGCTTATCCCCAATTCTGGAAATTCAGTCTGCACATGCTTGTATCACAACCCTATTCTTCACAACGTTTTATTCTCTACATGTCAGAAATAAATGTCTACTTGCCATCTGTATGCATCATACAGACATTTAGGCTCCTAGTATGGGTACAAGTAGCAAGTTAAAGTATGTTAATCTCAGAACTGAATTTTAACCAATAGGAGgttccatttattcattattatgtATGCATACCTTCTGTGTTTTAACATTGTGAAAAATCCAGGGAATCAAATACAAATACAGCAGGGTTCCCTGAATTCAACAAAGTGGGCCTTGGGTTCAGGCCTGGAGGTGAGATGGTGAACAGTTTGCCTTTGTAATCTTGGAATGAAGCACCTACCAGGGAGACAGAGGCATAAACAGACCTTTCAGCACAGCATGGTGAGAAGGAAAGGTCAGGTGCTGTGGGATCACAGGGGCACAGTCCCCAACCAAGCCCAGCAGAGGGTGGGCAAactcaaggaaggcttcctggaggagaggggcaTTGGGGTTGAGCCCTGAAGGAAGGACGAATAAAGAAAGACAAGGGAAAGCATGGTGCATTGGGGAATCTCCAAGGATTttgggaggagcaggagaggtGTGTACGGAGGTAGGAAAGAGATGGGAGTAAGCAGGGGCCTGGCTGCCAATGCAACAGAGCAGCTTGGACTTGGTCATACTTTCATCTGCTCAGGGCTGCGCTGGGGAGATATCACTTCCCCTGGTAGTGTGGGGGAGCCCAGCAAGACTGGATGCTGAGACTAGTTAGGTGGTTTTTGCAAGTAACTATCCAGGTGTGAGAACAGTTGCTGGAGCTGAGAAAGTGGCCAGGGGAAGAAGAGATGTTGATGGATTGATGGGAGATACTAAGACACTAGAGATAAAAGGACACGTGACTGACTGGTTACATATGGGGGTAAGGAAAAGCATGGCAACGAGGGTGTCCAGATGGCTGCAACAAGGAGGTTGTGGTACCAGGGTTTTGAGACAGGAAACCTGGGAGAAGCAGATCTAGTGGGAGAGGACACAGTGGTTTTGAGGTGCTGGTAGAATATCCAGGGAGTTTGGAACTCACAGTCTAGTTGCAGGCAGGGAACATAGTCTGTTCTTTCATATTggtctattttcttttctctgaaaggCAGATTTCtcaagaaatgtgttttctttcattattaaatGATAACAGCCGTTAATGGTTCTATGAAAAGCAAATCTGAGATGAACTGTTCAATATTATTGAGtagtgtttgttttaaaaaagatgggGCTTAGAAGgatataaaggaaggaaaatgaaagagagttCCCGGCTCTAGCTGCCCCCTAAACATTTCGGGGAGTTGAGTATACTAAATGTCAACTGTGTAGAGAGATTTAAAGatagagagggagggggaaaacatttccaggaaaaaagcactggggaatatttttgtttttagaaacttGATGATTTTGGATTACAACATGTGCTTAAGTGATTAGTAAGTAATGTTATCTGGGTCTCATTACCGCCGACATTTATCCACAAGCACTTACTCAGCACGTACTGGGTACTGGGTACTGGACAGCATTGACCAGACTGGAGACCACAGTGGGCATGGTTCTTTTCTCACAGAATTTAGAGTTTAACTGGCAGATTATAAATCCTGAAGGTAGCTTTTTATGATATGTCTGTCTGTGTCCAtttaatataattgatatacatcGTGAGGACAACTTACGATTCTCAAATCCcataataatgaacatttatgcCTCCAGAGGGCACTGGCAtgttcacagaaggaaaaacaagaaaggatTCAGTGCTGCCCTGACTTTCTGTCTGTTGAGGTGGGGCTGGTGGAATTGTTCCTCAAAGCAGGGGACTGGAACATAAACGGACAGTGGCTCCTGGCTGTTCTTGAGTGGCTAAGAAATAAAGAGTTGTGACTCATTTGCTCAAAAGGGCAAGGACAGAAAAGTTGAAGCTGAATACACAGGTTTTAGAGTTAGAGGGAAATTGGAACCTGGTACTGCCTTGAAATTTTGACTTTGAAAACAACATCCTCTCAGAAGCTGCACTAAGGCAGACTTTTGACTGTAAAGCCAAATGAgatcatttttttcaacaaaccACAGTTTGTTATTTCTATACCTTTCCTGTGCCTAACTTAACACGATCCCTCATTTTTCGGGGGAATGGATATCCCTGCTACTGTGTAGTCAacctcttgtttcttcttttttgttttctcctgatttcttacctcccttatatattttaaaattctccctcttcacttaatttcttttcctccattgCTCCCATTCTAAAATATTCCCTCCAGACCCAGTCTCCCCCTCTAAACTCCACTCCCAGTAGTTCCCTCCTTCACTGCTGAACTTGCAGAAAGCTGGGTTTGTAGAAGTGTgtctttttcttagttttctctaTTCTCATTTTCCATTCATCCTCATGCAGTCTGGTTTTCTAGTTATCTTAAGTTGTCTAGACCTGATAGTTCTCAGTTCTCACCCAGCTTAACCTGTCTGCGCATGGAACAGTGTCCTTGGCTTCTGAAATGCTTTGGCCCTTGACtgtgttgcttttgcttttgcttttgctggccacctccctcccacctgctctctgGGGTGCTGCCCCCTGCATCAGCACTGCCCCCTTGCCTGAGCCCTGGTCTCGGATTTTCAGTTAAGACATTTCCTGATCgcccctcctctgtcttctccacccTGGGCCTGCTTTCTCTGCCCTTCTGAACATCTTGTCTTGATTCATGGCATCACTCTCACTCCAGTTACCAAATCAGATCAGTGTTACTCTCATAATGTCTACAGGTTAGATCAAGTCTACCTTTTTTAAAACCTCTCAAATCCCACACCTTATTCTGTTCCCACTATCAGAGATACTTGGGGACACAGGGTTGAGGAATAAAGGTCTCTGTGCTTGGAAAGGCCACAGTCCACAGTGCAACTCTAAGTGTGGTCCAGGTACTGTAGTAGACcacaagctttttattttaatagattaattttcagagcagttttagattcacagtaAAAGCGAATGGAAGGTACAGAAATTTTCCATATACCACCTGCCCCTACATGAGCAAGGCCTCCCTCATTATCACCATTCCCCACCgaagtggtacatttgttatagcTGATGAACCTGTGTTGACACATCATcacccaaagcccatagtttacattaagatTAACCCTTGGTGTGGTACATACTATAGGTTTGGACTAATGTGTAATGACAGGTAGCCACCATTATAGTAACGTAcggaatagtttcactgccctaaaaatcctctctgctctgtctcttcacccctcccctccccccaaaccctgatagccactgatctttttactatctccgtagttttgtgttttccagaatatcatgttattggaatcacatagtatgtagccttttcagattggcttctttcatttaacaacaTGCATTtgagtttcttccatgtcttttcatggcttgatagctcatttctttttaatgctgaataatatttcattgtctgtacgtaccacagtttatccatttagCTAATGAAGGACATCTGGGTAATTTCCAAGTTTCGGCACTTATGAATAGAGTggctataaacatctgtgtgcaggtctTTGTGTGGATGTAAATTTCcagttcctttgggtaaataccatgGAGTGctgttgctggatcatatgtcagaggatttagttttataagaagctGCCAGACTGTCTGACAAAAGTGGCTGTATTGTTTTGCATTCCccccagcaatgaatgagagttcctggtGCTCCACATTATCACTAGCATTTGGTAGTATTTGtgtctggattttggccattctgataggtgtgtactGGTATCTCACTGGTATGTCTCATATCATCCAGCTGAAATTCACAAACGTGTGGGGCCCTATGACTGGGCCCGCCTGGAATTTTTGTCTCTCGGAGGTGTCCACATTGAGCCTTCAGCAACTTATCGGTTATAGATCAAGTTCTCCAAGCCTGGCACTGGAACTCATGCATTGGAATTCCTGCTTATGGGTTTCTATCCAGTAAGTTGTGGTTTTCTGTAGTCACCTGTCTCTCTccaattttagaattttagggGCAGAGGCTTGCCCTGTAACCTCACTTCTCCTGTGGAGCTCGAAAGATaggttgatttttcagtttgctcagctttttattgttttagagtGGAGTTGGTGACCTCTAAGCTCCTTACATGTTGGACCAGAAACCGAAAGTCCACAAACATACTTTATCCGACTGTGACAAGATAAATACCAGGAGAGAAAGCATTTAGAAACTTGAGTAATTTGACATTGCCATGATACCCAAGTGcatgattttatgttttataaaaatattggtcTGCAGAAGCTTggaaattagaaacaaaacaaaacaacaataaacaattGGCCCTTCACTACAGGTGGAGAAGCATGGGACTAGGAGGCAATCAAGAACATGATGATTTGAGGTGGGAATTTACACCATCTTTGCAGTTATCCAGAAGGTGAGCTGAGATCCCTGGGGAAGGAGGTCAAACTGATTAGGGCTTTTCAGAGTGGACGTGGTGGGGAAGATACAGTGGCATGTGGGCGAGCATAGACATACCCTACTCCGTACTACCTCACCCCTCTACCTTTGTAACATGGAGATCAGATCATGGCACGTCTTTGCTTAAATCCTTAGAGACATCCTTCTGCCCAACACTTCTCAACTTCTCGGCACCATTCATAAGGCTGCTCACCCTCTGACCAGAGGTCCCTGTAGCATCGTTTTTTCCCACTCCAGCCCGTGCACCCTTCTCTCCCAGCAGCTGGTTTTCCTAGAGTAGACCACAGCATGCACCGCCACCTCTGTTCTGTACCTCTGTTATGCATATGCAAGGACTCTGCATCAGCAGCCTCTTCTGTTTGAAAGGTCTTCCCTCCTCTCAGTCTCTTCTGGTGAAATCCCACATATCCTTTAACATCATCGTCTTGGAGGTTTTCCTGCATCTGATGTCAAATGCACAGGTGAGGCGGGCCTAGGTGCTCAATCAATGTTGAGTGAACATGGAGCTAGAAGGGGAACTTAGAGATTCAGCCACACCTCTGATCATCTCATGTCCTTGTGTCTTCACTGGGCCTCTCACAGCTGGAAAGGGGGAGGCATTTCAGGCCATTAGGTAGGAGGAATTACCAAATAGCACTAAAACCTGCATCTGAATgcctcagggctgggggagacTGGAGAAAAGGCCATTCAATATCAGGGGATGAGGAGACCAGCTTTCCAGGATGGAGGCAGCTTTCCTGGGGTTCCCATCCTTCTCAGAGACAGAGCCTCTAAAGCAGGGCTTGGCAAGCTTTTTCTTCAAGGAGCcagataatgaatatttttggCTCTGTGGGCCAGACGATGTCTGTTGCAGCTATTTTGTCACTGCAAGGCACAAGCAGCCATGGACAAGTGGGTGTGGCTGAGTTCCAATGACACTCTTACAGAAGTGTTCCAGTAACATTTATGAAAACAGGCTGCAGGCTGGATTTAGcccacaggctgtagtttgcccACCTCtgctcaaaggaaagaaaatgtccCCTTTCTTCTGCCCTGTCTCTAAATAGCCTTTCGAGGCCTCGCTAAGTTTACCTTTATAGTTTTGaaattcttagatttttttttcccctccaaaataGCATAAACAATGAGTCATCTGTCCCTACTTGCCAGTCAGTTGCAGCAGCCTGAAACACCCTTTACCAGTGGAGTGAAAGATGCATGATCTACACTGAACATGTGGAAGTTCGTAAAATTAAACCTTAACCCAGGTCTCTATGAGATAGACACATGAGATTCCTACTGTGAACTGAGAACTAAAACCCTGCCATAAAGTGGTTTGGTAGTATCCCTAGTGCACTTCCTGTGTTTGGGTGGCTATGTGGATTTGGGACCTGGAAAGTGGACTTAAGCTTTTCATAAACTGGGGTAACCCTCGGAGCAGAGATGATGCCCATGTCCATGGCCTACTATACTGTCTAGGATTCACAGCCTATGGGAAAATTTGCTAAGTTACTGATGTCGaaatgttgcagcaaagtgtgttacagctcagttgtgacagcaacctggatctgggcgagagatCAAGCAGCACTCgaagagttggagaactcaggtttattacaccagcgggcccagaggagttaacactccaagctctggaccctgtctgtaggtttacacagccttttataggctaccagtctagactttgcaacattttgtaacatcatatgcaaataagttataacaaaggtgactaattatgaacaagctttgtagaaatggaccaatcaggaatgagagaaatggaccaatcaggagtggtagaaatggaccaatcaggagtgagctccatgcaaataaagcactacaaatggaccaatcaggagttagctcaggaaaccaatagaattttaggggtaagttccactttcttagaagtaaactgtttcagaggcaaaaagtgagataatgccactgggccagggagcCAGATGGTGGTGGCAGGAGAgtaatggccctgcctgggggtcttgCTGGTgtttttcatggggcttcccacctaTCTTTGCGTTAATAGAGTGGCAGACAATACAGCAATGCATACATTAGGCCAGTGACATTAAAGGTAAGTACACCTACCTCAAATGGTCCTACATTCTGTAAAATGCTCAAACCAAATAGCCatgtaaattaaaatctaaaagagTGGGGTTACTAAAACCCTGACAGCATCAGAACCAGGGCAGCAGGTGAAGGAAGCTTGCTATCAATATGACAGCCTCTGTCACCTCCCATACCCCAAAGAGGCTCCACAGAGGTCACCCTGTGACTCTTGTTCCCCAGAGTCACAAATCAGGACGTCTTTTGTGCCTGACTGAACAGGTTGACCTAGTAGCAATGCTTGTGCCAGGGAAGCTGTGAGGGTGGAACTTGGACGTGGGCCTCGTCATCTTCTGGTGCTGTTTGAAGTATCCTCAGGTTAGCCACAGTCTCCTACTCTGAAAACTAACCAGTATGGGACATTATTCACCCAGCAGCTTTTGCCCCCAGATGCAGTCAAAATCAGTGTGTACTTTACAGCCATCAGTGGAGTGGTTTCCTTTAAAATCTcaaattctttccctttccttatgTCTTTGAGGTTTAAACACCCCCAAGAAAAGTTGGTGATATTCCTGATATCTTGCATCTTGTATTTATGACAACCAGACCTCTTGTAAACTGTATTTCAGATAAAcacaatttattgagcacttactaggtCATTACAATGTGAAATCACATAAGAGTGGTGTAGACTATTACCTGATCATCACACAGATGCATAAGCTAGGTTTATTCTCCTGCAGCCTCTTCTTATGAGTGTAGTATGTGATTATTTAGATTTTATCAAAAGGTTATAAGTTagcaattttatatttcttttctcatcGTGTACCTTTGGACatttagttttccattttgaaaagtcCATTTGGAATAAATTTATTGTCGGTGTTGAAGAGGGctagttttcatttattctaatgtTGTATACTTCAGTGCTTGAATTATTTACTATGGGTTTGGTTATTGAcctattttatttgtcattttatgatGTACATATTTTCAGTGGAGACACTTAGGCTaagctatatttatttataccacAGTACATTAACCATAAAACTATCTAACTACAGTAAAAAACCACAATAATCTGGAACCCTCGGGGAGTGGGTTATTCAAGATAGCCAAGTTTTAAGAATGAACATTAACTATTTGTGATGGAAACCTTTTTAAATGGATCATACTTTCCCCTCTTGAAAATAAGATAGCGAGTCTCACTGAAGTTTGATAACAACTCAGTAAGAATTGCACTGCATAACTCAGCTCTCGTGAAAAGACTTGgatgtttttggatttttttcttaattctcatGGGCATTCCTTAAACCAGTCCTCTTTGTTCAGTTGCTGTCATCTGTATTTCTCACTCACCTTCAAATTGCTGCTTTATACATCTGTGGACCTGGAGACCAGGTGACGGAACTCATGAGAATATAGGCAGGCATGAGAAAGGGCTTCCGGGGACCCACTGATGGGTCAGCTACCACAGTTGTGTATTGACTTGCCAGCAACTGTTGTCTCCCTGAAAACCCTTATTTGCTAATGTTTCAGTTCTCTGTGTTCTATCCAGTAGGTGGTAAGACAGGGCAGATCTGCTGTACCTCGTCTTCCTAACATGCTTCTCTACTCTGCAAAACTCTGCAGCAGCTGATTCCAGCCAGGACAGGGCAGCTCTGGGCCTTGTCTCTAGGAGCTAAGGCAGCTAATTGAATactcattagaaaagaaaatcaggttTAAAACAGGCCTCATTTTAAGTTCATAGTTTATTAAATGATGCCTATATTGTGGTAATACCCTTCaaattctccttctcttctttatcctattgcttctttttcctcttttttacaTAAAATGCTAAATGTATGCTCTTTGGGCTTGTAATTTATTGGGACTAAAGCTAAAACCTTAATTCTAAAAAAGCATCTCTACACACattactgaatataaaataggtaaacaacaaagatctactgtctagcacaggaaactgtattcaatttcttataataacatataatggaaaagaatctgaaaataaaatatatatgtatgtatatgtataactgaatcactttgctgtacacctgaaactgacattataaatcaactacacatcagtaaaaaataaaaaataaataaataaaaaagcatctCTAATCAAAACTGCAGAATCCttaataattatgattttttaaatacacattattatggatgtgtttcatttttttttactttaggtTCTTTGCAcaaaaattgaatgaaaaaacCTTTATTAAAGTTGCTGGCAGGTGAACATTAGACTGGAAACTTGGTTGTTTGACACTTCCGTGGTGCTTTGCAGCTTCTCCAGTGGTCTCATCGTGTTTTCTCACTGGATCCTCAGAACAACGCAAGGGTTATATTGTCCCTCATGTCAGAGCCAGGAGATCAGAGGTGCAGAGGTGTCCAGTGACATGTTTAAGGTCACATTGCTGTTAGCCTCTCAGGATCGAGCCCAGATCCTCTGCTTCCCAGACCTGAAAGTCTTCCCCCATTAGCTCCTAAGTTATCATGCTGGTGAGTACGAGATGAGACTTATTAGTTTACAGTGGGCAGACACTTTAGTTCTCAGTGTGCAGTTGGGGCTGCCAACCCCTCTGAGTATTATGAAGTGTCAGCTCATCCAGAGTGAGACGAGGGGAACTTGATCAGCCTGTGATCGAGGGGAAGAAGTACGTCATCTTCAACTAGCCGTCACCCTGCTGCtgcgggaggagggggaggattGCCCATTTGGGAGCCGAGGACCAACTGGTCAGTCGTGTACAATAATTTGAGGATCCTGGGACTAAATGTAATAGTTTTAGTTATTACTGTAGATAACTGTCCAGTGTTTTAGAGAATCAaatattgttttgtttagttGTGACCACATATTAGTAGTCAGTTGGTGAAATTATTGTTTTTCAAAACTAGGTGCTAAAGtgtttttagaatttattctGATCCATTTCTTAATACAAACAGTATCTGTACCATTTGTATCCAAAGCAcaagtttcctttatttttactttcagattTCAGAATATACCTATTTTGGAGCTTGAAAACATTAGTTTGGCAACAACAGTTTGCGTGACTGgatttaatttgttatttacaAACTGTATggctttttgttttaagaattgTTAATGATAGGCTATGTTAAACCACTGTTTTTGAAGGtctattaaaatgttacattccaaatagaataatatttaaataaacctttaacaaatttgtatttttcttttcatttatgtgtgtatgtgtttgctgACGGAATGGAGATTGCTTCTTAAGAAGCCAACCCAACCCCAAATTTCTAAAATGCTCTCATTATccagtttagtttttaaaaaattggaaatttagGCTGCTCTATTTATATTAACAGACTGGTGCTATCAAGTTAATCCtgtaattaaaattcatttctcaaaGATGTGGCTAACATCTTGGaaatattaaagatttaaacCCTAAAAATTGGTATGGAAATGATTAAATTTTCcctattattttttccttctctcccagagtgattcatttcactttctgtgtcttcttccaTGAACCATAATGCTTCTGCAGAGATTCCTAATTCAGTGAAACCTGTTTAGGGGATCATCTCTGGTAGAAAGACAGCTGCCGAAAAGGCATTCACTTTCTGAGCATGGATTTTAGATTATGGCCAATCAAGACGCGGAGCTGGAAGAACACCTGTTAAAAGACGTTTTTCTACAAGGAGATGGTCTCTTTAATCAGATTTCATTGCACTTCagaaaaagattcatttaaaaatgtgggtAAAACGGCATCTGCTTTTATGATTCATCCTCACCAtccttttaaggctgaaaaacCAATTTAGAGTTTATTTACGACGTTGTTCTACGGTAGAAGGAGAAACTTCAACAGagtgtttaaaaaggaaagactCCCAGACATTTGATTTGAACCTTTCTCTTGTGAAAAAAAAcctaagaatttatttttgaatttttgcgTGTGCATATAGACAAATGATAGGAATGCTTATGGGCAAACTTTAAAAAGGTATAAGCTTAATTGTGAATATGTGtaatgaaaactggaaaactttCCTACGCTGAAATTTGTTGCACTTATCTGTGAACAAGGCAAAGTATTATTGATGCTGACTGGTGTTTAGAGAAACTACACTCAACTTTCAACTCTGCTCTAATTAGAGGCACCGTTAAGTACTTAAAACGCAGGTACTTTCTCACATCTGCAGACTATATTGAGCGTAACTATTAGAAGACAAACTTCATTTCGCAAACTGTAATTTAATGGTGTAACAGTGTGCAGACCGGAATATCGGttgattttcaaaacattttattctccACCAATTAGCTCAGGTCCTAGAGGGCAAAATGACAAGAGGTTACTCGTTCACTAtgtatttaattaattgaaaaatgcTCAAATTGCTTTTACAGTTGTTATTAAAAAGTAGAATCAGTGTTGCCAGTGCTCAACCATAATTGGAACCCCAGGGAATTAAATATTTAGCATTAGTAATGTAACAGCACTCAGGACACAAATTTCTATTTTCGTAATGAAAGCTATACACTGTGAGTGCAAGCGGCTGAAACGACAGCTGTGACAAGGGAAACGGCCTGGGATATTTCAATAAGACACCTCATTGGACAGCAAGTCTTACCACATTACTCGTTGGTAAGAGATCTGCAAGTTTAATTCCCAGCAGAAACCCGTTACCCAAGTATTTAGATTTGTAATTGCACGCAGGATGAACAGTTGGGCAAATGTCTTGCCGTAAGTGGCTGAAATGTATAGTGCTTTCTAAATAATCCTATATAAGTTCATGCctttgtcaatttaaaaatagcacGACTGTCAGGGCATAAAGatcttgtttgttttaagtttaattacattttaagcATAAATGCTTTCAGTGTTACAATGAGAAAAGTTGGATATTAGGCTCTAATGAGCTACAGTGGTAGACGGCTGAAAACCCTAAGGGATTCCCTCCGTAAATTAACTGGATAAAAGTACTTGGTTAGGATATTGTTGATGGCTGAGCTGTTCTTAAAACCCAGTTATAGAGCTGTCTCACTCCTGTTTTCATTCTAAACAGGCCCTGTAGAGACTTTAACAGCCGCCTGTTACAACTGTTCAACTTgtcaaaatgtattatattacTCCACTAATAAATAGTATTAAAGGGCATTTgacttcatttctccttttcagaGGGGATCAGGTTGATAAACACAACTGGCTTAGATCGgtctcattttaatttgtttatgtcAGCTAAGTAATATTGAAGGTCCCTGGTTTGAATTTGGCAAACATTATTTTCCCGAGGATgtaaaacttacaaaatattatGGACAATGGAAACAATTAACGGAGACTATTTATGGTAGAATCTTTGTCTGTGAAAGATGTATGACTTTAACATGTTTCCCCACGTTCAAAGTATTTGAAAAACCGTAGCAAAACTGACATTCTTTGAAGCCTAACTCGACTAATTCCTTTGCCAATAattctgtcttttctttaaagaagaaaaagttgcGGGACTTTTTGATTTCacaattttcttttgcttatcttTGTCTGAGTCAGAATATGTTTTGAGAACATATTCTAACTgatcatgatttcttttttcttttattttctgttatttttagaaCACAcgtgcttatttttaaaagagagagaaaagaaaaatgtagaaatttaTTTGATAAACCCTCCCTTTTACCCTCTCCCCCAATCCTACTTTCCTGTCTGGTGGTCACCTTGTTAACAGTTTGGCAACATATCCAACATCCATAACATTTTCTGTGCATATTATATGTACATGTGCACATAcagatgtgtatttatttatatacatgacACATTTTGGATCATTCCATGAAGTCTGTGATTTGCTTTCTTCCCCCACTTATCATagatattttcctattttggTGCCCAGATCTTCC |
| LNC 000062 | CCCACCCCGGCGGGCGGAGTAACGGTTTGGAATCCCCCAGAGTCCGCGAGCGACGGTCTCCTACCTCCTGGTCTAGGAAAAGTGTAAACACGCGCGCTCCGGAAGGCGTCCTCCAGTGGGAGGTTGCCCGGGGAACAGCTGAGCTGTGTGAGGCTCCGAGCTAGAAAGAAAGGCTCTATGCTTATTGTCTGCATTTCGCTTTGTCGTGACGAGCTGCGGAGACCCACGCAGAGCAGACTTGGGTATCTGCCCCCGTTCCCACCTCTTAAGTTGCTAACAATTGAAACCCATTTCTAACGCTTGGTTAGCACTAATTTTCAAGGGAAAACGCTGGTGGCAGTAGAATCCCTCCCCAGAACATCCCTCCTAAGGACTGGCAGCAGGGCCAGGTGGAGGCCGGGTGCAGCGCGGCCGCTTCTTACTAGAGGACAGCGACACTAAAGTGCATTGTGGAGCTGAGGACCGGAGAGCGCGTTAGGGCTGGCCGGAAGGGTGAAGTGAAAGAGGAACGGCCTAAAATGAGGAAGAGACAGTTATCACCACCGGCAGTACCTAGTTCCAGTTCTTGGTCCCAGGGGTTCGTGCTGGCTGCCGCTTTCATCGTCCTCAGCCGAAACAAAAGGCAAGTGCCAGAGAGTGACTCCTGAGGACCTTTAGTGCGGTCTGCACCTCTCGGTCTTGGCGCATCTGTGTCCACTCGCTTCTCCAACCCTTCACACTTGATGCTCTCGAATCAGTAGAAAGGAGCACAATTAGTGGAAAACACAAGCTCGGGAGTCAAGGGATTTGGGTTCTATTCCTGTCGTCCCTCGGTGACGACATCATTACCTTGTGTTATGCACGCTTTCTGTGCCAGAGTTCCTAGATCAATCAGATTATTGTATTGACCTCTTATAGAAGTGGTTTCATGGGGCTGAATTACGAATAAAGTAGGTGTAGCAATGGGTTATATCTGTTCGTTAAACAGTAAGagctttaataataataataaagagcagTACTGTAAGTATTCTGTGTTATCTGGTGAGTGTTTTACTGGGATCTTTTTAAAGACTTTCCCACTATATCTGCAGCATTATAAACAAGTTTTTAGGTACATTCACAGCTACGGCAATTGTGAATCAAAGGAACTCCTGCTTTTGTTAAAATGTAAGTTTCTGTATTGCTAAGTGGTTTTTCAAGATGCTCTCATCTGCTgcgcattttttttttaaattttgcaggCTGGTGGAAGCTTAACCTTTCTCCCATACCTCCCTATTTATCTAAATGCAGTGCTGATATGATCAGGCATTTTTCACCAATTTGGGGTATAAAAAAAGGTCGCTTTCACCTAGGAggagctctttttatttttcctatcctCGTATTTAGCCTGTCAGCCATTGACGGAATTGATGCAAATCCAGGTTCAGAGTAATAATATTAGTGTCTGCTGAtgctaatatttaaagaattagaattaaaaagagcCTTCAAACTGTGGTTTGCGTAATTTAATATGTAAGTCAGAAGTTTTGTCAGCATTTTATGGTGCATCAAGTTATGAAAATTTACTGAGAGAAAACTCACTTATTTTAGATTTCCTGTTGATTTGTTGAATTGTTTGACTTGACCTCTGTTGCTCTATGTATTGTTTTAAACTCTATATTCACCTCCCCCTTAGCTGTAGTTGCCTCACTTTGCCAGTGCAGTAGCAGTATGACACTAATTTCTTCACCTAAATTGATAAATGAAACAGTATGAAGACTTACATTTTATTACTGCCCTCTGGTATCCGTTGCTGGTAACCTGGGCTCTGTAATTGGtcacaatttctttattgaaAGTCGACATtcgtatttattttatagcttggTACTGTCCTGaggtttataaaaatgaaatgtcatcGTAATGGACGATCTGAGCACTGAGGTGCGTATTTTCATCCTACTGACAGGCATCTGCTGGCCTGTGCATTATAAGCAAATGTTCTTCAAGGTGTTTTGATGGCTTTCCATTTGTATAGCACTGTTCACCTATGAGGAAAACAGTAGGGGTACCAGAGAGCTTTCCCCAGGAGCCTGTGTGAGACTCACCAGTACCAGATGAGGACGGTAACCTTCATGTCCTTCACGCTCTTTCAGTTGTATCACGCTGCAGGGGCTAAGGCTGCAGGAGGCAAGCACTGCAATCCTGCAAATAATGGTCCCTACAGCCAGGACCATACCAGCCAGCctttatagaaataattaaaaacatataatgaAATTCTCAAGTGTTTCAAGGGAATGCAGTGCTACCTACTTGCAGAGAAAATGACGAGCAGTAGGATAAAGGTAGGCTTTATGGAGGGATATAAGATGACCCTTGATGAACGGGTCAGGATTTGAAGATAAGAGCAATGAAGGAGGCATATCAaacaggaaggagcaggggcaCAGATGTAAGAGTTGTTATAGCTTCTACAAAGGACAGTGATCAGCTTCAGTTAGTACTGCTGATCCATGTTTGGAAGtactgaaaagtgaaataaagtatGATTGTGGAGAACCTTGAAAAGTAAGATTTCAGAGTAACAGTTAAGAAGGAGCATATATACAAAACCTAGTGTGGGGGGGaggtggatgggaagggacagactgggagttcgagattgtatagcaaagggaaatatattcaagttcttgtggtagctcacggggaaaaagaatatgaaaatgaatatatgtatattcatgtatgactgaaaaattgtgctgtacgccagaaactgacacagcattgtaaatcgactatagctcaatttaaaaaacaaaaaacctagcaTGATCTATTcagcaagatatattcaaaaacAATGTGCAGATCTGAAATAGTTGACCGAGAAGCGTGAGACCTCTTTGATGGCCAAATGCCAGCAAGTATGAATGAACTGGTAATGATCAGGGTGGCATCCACCACACATAAACAAGTTTGTTAAGTAGCGGTGGACGGGAAAAATAGTAGCATAGAGTGGTCAGATATATGCACTTGGGAATGAAAAACTGTGGGTTCCAAGCCCAGCACTTCGGTTTACCAGTTATAAGACTTTAGATTACCTAATCTTTgtgattgacttctttcacttgtgAAAAGGGGATAATGATAGTACCTACCTTTAGGGTGGCTGTGGgaatgaaatgttattttctattgcACAGTAAGCCCTCATTAAATGGTAGTTAGTATTCATTGCTTCTTGAGGTGACAgtaaggattacatgagataacgTATAGCAGCTGGCACATTATTGAATGAGTATTGAATGTTAGCCGCAATGTCACTATTACTTGTAGGTATGGACTGGAGATAGGTTTTGAAGATAGGTTCAGTAGGGAAGCCGTGGGCCTGTTAGCATCCTGATGCACACAAAGCAAGCCTGATGTTTAGCCAAAATTCTTTGACTTCTGTATATTGGAGTCAGTTGAGTGACTGGAAATGAAGCTCAGTTAGCTGACTTAACTCCAGCCCCACACAGTCCCAGCTTCAGCATATTACCACAGTATGGCAGCCAGCTTTCCAAACACCGTCAAAAACTGGGCAACAGACAGATTCCGGGAATAAGAAGGAAGTAAGACACAAGGACTGAGCACTGCTGGTGGAAGGCCTCTCCCTACTCCCTCTGTACACCCGCGTGGGCAGGAGTCTGAGCCGGAGAGGCCCTGCAGCCTGCCTTCTCTTTCGAGAAGGAAAATTTGGCCATGGATATCCTTTGAGCTGGACAGGCCTGctcttccctccatctcctctgctcaGAGATAACCATCTTggtttttctctctgcatttctaTTCAGCTCACTCCGGAAGGAATGCTTAGGGTCTGAAATTGCTGTGAGCAAGCAGTCTGTGATACTGTTTCCTAGACTTGAGAAAGCTTTTGTCCAAGGGCTCCCTGACCCTCACCCCAGCTCCCACTCCATGCCAGTTGGAAGGGGCTTTCCCTGGTATACTTGGTTTGTAAATAGGCCCTGTTAGAGTTAAAGAAATCTCTGTCACTcaatcagaaatatattttatgtcatAGCCcagtacacaaacacacacacatatacatagtgTCATGAAACTATACTTACCCTTTCTACAAGCAGTGCTCTCTGctatttctattctgttctgtccttaaaaaaaaaaaggggtggtTATGACCCACTAAAATGTTAAAGTACATTAATGGCTTGGAATTTAAGTTTGAAACCAGTGCTTTATATCTCCCACAGTGTTTTGAAGAGGTTATACTATTCTAGAAATGTCTGAAAGGAGGGATAAATCTTTGCACTGGGAGGACCATGGTGTTGGCTGCATAAAGGGGTGATGAGGAAGAAGATAGACATCCAAGATTAAACAATACCAGGAAACTCAAGAACAAGGCTACTTGGAGTTTCTCTCCTCCTCAGAACTGGGgtttcaaatgaacaaaaacaatttCTTGACAGTAGATTTAACACCCACCTGTCCCTCAGCTGAGAAAAATACTAAGCATGGATTTGCAATTCTTACTCCCACCTTAAAACCTACCTACCCATCAAGGCCCCTGCTCAGATGCCACTCTCCCCATCAAGGCTTGCCTGACTCATCCTTGCAGTGGGTGACATGTGGTAGAGAAGGAGCGTGAGCTTTGGAATCAGGTAGAACTGGATTCACATCTCCACCCTTCCTATATGACACTGGGCAATCACTCAAGCTCAACTTGtatatcagtaaaatggggataataaaaacTTCCCTTAGAGAGTTTTCCTGAAAAGATCAAAGAAGATAACATCCTTGAAGGCTCTTTATTGCTTGCCAAATAACACGTCTTCCATAGAtgttcctttccttcattcctctTGGTAATCACTGTCCCTGCAGAGCATCCAGGCACTATGTTTCCTTTGAGACATCACTCTCTACTTCATATAGCAGCGGCATACTTACTTTATCCACCCTGTAAGACAAGCTCCCAGGGGACAGCGTCCATTGGCCATTCATAGTCTCATAATACCCAGTAGTATAACAGAAGGCGCTCACAGAATACTTGTTGACTTACTGAAGAACGTCATCAAAGGTAATATGACCCTGTCAAAGAAACGTTTAAAGTGCTTGCACAGAAATGAACGTGATAACTGGATAAAAGCttacaataaaaatagtaactgaTAGGATGGCACTGACCACTAAAATTGTAAACAATCAGTGACAATTTATTACACCAGTATTCGTAGTTCATCCAATAGAATGAGCAAAGAACACTGACTAGCAGAAGACATTAGCTCATATCTTCAAATGTTAAGGAACTCAAGCAGCAGAGCACACCTGAGCTCTTCTTGAGTgagctggagaggaattttgcctcaGGATGAATCCTGTCTGTCATTTCTAGAATGAGATTTGGACTTCAGACTTCAGAGTTGATACTGAAATGAACTAAGACTTTTCGAGTTGTTAGGATGGCATGGGTGTATTTCGCATGGGATAAGGATGTGAATTTTGATGGGCCAGTGACAGCGTGTGAAGGACTGAATATTTACATCCttctaaaattcatacattgaagtcCTGACCCCCAATGTGgtggcatttggaggtggggccttcagAAGGTAATTAGGATCAAATGAGGTCATGAGAGGGGGCCCTTGTGATAGGGTTAGTACCCTtggaagagaccccagagaacttgctctctcttcctccacatGCACCCCGTGGCTGGGAAgggagctctcaccagaacccaaacAGGCTGGCATCCAGATCTcggacttctagcttccagaactataaggaaataaatttccattgttcaAACCACTcactctgtggtattttgttgttgCAGCCCAAGCTAACCAATACCTCTAGGAATGACCTGATTTTCCTGAGTGATGAAGAAAATCCTTGAGAGCACCATGAATCTGAAGGCTACTCAGCAGAGAAAAGATGAGAACTTGTGTGCCCTGAACTTCTCACGGAGGGGctcttctgtcctctcccttcATCAGGAATAAAGACTCTAGTTTGTCAGGGCAGAGTCAGTGCTTTCTAatttgaggggaggaagggaaagggtcAGTCTTCACCCTGCTTCACCTAGCCTATTTATTGTGAATATTTGCTTATCTCTTGCAGtgaaagacaataaaaatgatgGCGTGGTCCCCCTGCATTCTTGCCTAGGGCACCAGAGAAATTCCGAACGAGTCCTGTCTGCCCAATGTCTGCTGCTAGATGTTTCTTTGGCCCCTTCTTGTTGCCCCAGGAGGCAACTGAGCTGCTTCCTTGGAACCCGTCAGGACTTGTCTTCTTTAGTCATCTCCCAGGTGCTAAACCACACCATCCACATCCACCCACTTCCCAATATATTAATGCGTGGACTCTGGAATTTTAACTCTGCCATTTATTTCCTGAGCGGCTTTAGGCAACTCACTCAAGCCTTCCTTGTCTCGGATTCCATattctgcaaaatggggacagttgtttccaccttttagtGTTGTTTTGGAGATTAATGACTTTAAATAGATTAGCATCCCATCTAATGCCGTGCCCAACAAGCGATAAACACGCAATGATTGTCAGCCATGGCCACGTTCATCTTTATCATCACCTTTGCCAGGTAGATACAAATCCTGGACCCAAAGCTGTTTAGGTGATGTCCACTAGATGGCACTAGCCACAGGTAAACAAAATCTACGCAGTGTGCATATATAGGGATAAATATATTGTGGCCCCAAAACTAAGCATTTGCTCCATGATAATTACCTAAAGCAGTTGTTTGAGAACAGCTACTAGTCAAACATGTTCTGGTTTGATTCCCAGaccccaggagattctgatgcaggAGGAtcagcagagaaacaaaaacgCTCCCATTAGGTTATCATGGAGTGCCAGTGTATTGTTCAGTCAGGTCTGGCTAACAACggaggtttttgttttgcagaaatgTGTTTCTCCAGCTTTTGTTAGGCTGAGTAGGGTGTGATTTGGAGAAAGACCTTGCTGCAGGCCTGAGATTTTTGTGTGCTAAGGGAGGAAACTTTTGCAGGCAACCAACCGCTGACAGAGACAGAAGCTTGGAAGTGCAGAAGAGTTCAGACAGAGCGTTTTGCAAGTTTTTCTATGTGATAAAGAGTATCACCAGAGCTTTTGCGAGTAGATGTGCTCTTCTGACATCTGTGAAAATGATCTCAAGGTAGAGCAAGAACAAAACGGGCTGGACATTTGAGTTACAGCAccttcttacttttctttcttgttactCCTTTCTGTTATCCCTAAGCCTTCAAATGGAGTCTTGTTTAAAATATCTTAGTTATACTGTAGGGAGTTAAGGGAGAGATTTGTGTCCACCTTTGGGCTGAATTATTATACCCATTGGAACTGAGTTACAGAAGTGAGGTGATAAAGATTGAAACGCGAAAGCACTCAATGGGAATCCTCATTGATGTTTAAAGAAATCAGATAAAAGCCTGGAATCCCTGTAGTTTCCTAGGATGGAAGGTTTTGTCGTTTGGGTATCCAAGAGCAGGGCAATCCTAGAAGACTGGCCATGGTCATACCCTAAACCAGCTAAAGGTCTGGGGGCTGCAGGCACATCCAGAACAGGGGGAAGGTTATAGAGCACACCTGGGCGGTAGTGATGAGAAATGCAGACAAACGGGACAGAATTATATTGGTTAAGGGGAGAGACTGACAAGAGCAATCTGGGGAGTTGTTTCTGAGCTTtctgtttagaaagaaaatagagcCAGTGGCAGACCCACTTCTTCAGCTTCCTGACCCCCTACCTCCAATTCATCTTACCTCCTTTCTACCCCAGAGGAAAGGGAATTCTTTGTCCTTATATATGCTAGATCTCTTCTGTCCAGACTCCATCAGTTATCAGTTCCAATTCTTTCATTGCTGGCTCTAGTCTCtagacatatttttttaaaagttcaagtcCGTACCTTTTGAAACAAAACTCTCTCTTCATTTAGCATCCTGTAGTTACTgtcctgttctttcttccctttgcagCCACGCTTCTAGAAAGAGCAGTCCCCCTGGCTCcactccctttcttccctttctgttccCCACCCTGCAATCCAGCCATTTTGCCCACACCCACCTGCAAATGCCCCCACGGAGGTGAGCAGGGAAATTCTTCCTGTCAAAGTCATTGGGTGGTTATCAGCAGGGACTCTCAGACAGCGTGCAAGTATAATTACTGAGCTCCTGCTGTGTGTttgacactgttctaagcacagtGGTGTCTCAAGGGTGCAGTCAGGTCCTGCTGCCATCAGAGTGACCTGGCCCCGGGCCTGGGCTCGGGGTGTGGGGAGCCCTGCAGCTTGGATTGGCGGGGCAGCAGGCTCTTGTGAAAGCGCCAGCCTTTGACTGCCTTCTCCGCCTGCATGACGGTGTGAATTCCAGACCGCCTGCGCAAATGTTATTTTCATCATATTGTTAACCACTCTTTTATCAATGTAGTATCTTAAAATTGctgaaaatgtgttattttaggAGGAAATGACCACTCCCCCAACAAAGCCTGCCTTCTGTCCTCTCGTAGCTTGATCTCTGGTGGGGTGAAACTCACAGTACCCATGTAAACATTTAATTTCAGAGAACTGTAAATACTGTGAAAGAAATAGAGCAGGACAACATGATATAAAGCACTTAATAAGCTCTGAAGGCTCATATCCCCTCCAAATTCGTAAGTTGAAATCCTTACCCCAAGGTGATgattaggagatggggcctttggaaaGCGATTAGGTCTTAAGGTGAAGCCCTCATGAGTAGGATCAGTGCCCTAATAAAAGAGGCCCCACAGGGATGCCTTGCCCATTCCTCCCATGTAAcgacacagtgagaaggcaccTGCCGTGacccaggaagtgggccctccCCAGACGCTGatcagcctccagaattatgagaaagaaatgtttgttgtttgtaaGTCACCGAGTTGATGGCTTTTTTGTGATAGCCACCCAAACAAACTGAGACCGGCAGCTGTGGCACTGGGAGAACTGGGTTGGAGTATGGTCAAAGAAGGCCTctttgagaagatgacatttaagtTGAGACTTAAAAGATGAAAAGGTTTAGAAAAACCTGGGGGAAAgcctgccaggcagagggaagagtaaATGGAAAGGCACCGAAGAACTTGGAATGTTGGGGAACTGGGAGGAGAGCCATGTGCCTGGAGCTTCAGGACATAACAGTGGTTAGAAAGGAGGCTGGACGAGGTGGGCCTTGTTTATCAGATGGGAGATGCTGTTGGCAGGTAGAGAGACCCCAGATGGGAAAGCCCATCAAAGTGTGGGCAAGCCCCCGCAAACTTGTGGGTCCTTGCCTGCCGTTGAGAAAGAATTCTCACTAGAGGCAGAGGGACAACATCGGGGGATGGGAGTAAAGACCTGCTTTATTGatcagagagagaaggtggggggaaggcaaaaaaaaaaaaaaaagaaaaagaaaaaaacacttgagCAGGAGCCGTCAGCAAGGGAGCTAGATGAGACAGCTCCAGCCCCCATCCGGCCACGGGGCCTTTTATTGGGCAGCTTTGCCATCGTGACCATCACTCAGGTGGTGCCAATCATTTTCTGTTACTGGCTCTTTCCTCCCATGGAGCTCAGCCCAAAAACTTTGGCAGATGGaagagagcaaagaaagagaTATCAGGATATGTCCTTGGGATCAATGCAGCAGTTGTGGCGTGTTGTATGGTTACGTCCTTGGAATCAAGGTGCCAGCTCTGGGAAGAGAGGAACAACTTGTTTTTTAACAGCCTGGCGTTTTCTTTCCCTTGTTGTTAATCAGGCAGGGTCAGTAAGCCTGCCTGCTCACCTCTCACAGTCACTGGAGCAGGTGTAAGTGCTAATAGATAAGattcaggagagagaggaacTGAGGTGCAGAAGAGGGTGGGACTTCACCACACACATGGAGGAGTTTGCTTTGATAACAGCAGAAATGCACATACCTTTTAAAGCTTTTCCCCCTTACTTTTCACAGTGTCACTCCtgattctctgccttcctctctgatTCTTCTCAGTCTCCCCTTTCATTTCTTCGTCAGCAGGGTTCTGTCCAACTATCCTCTTTACATCTTCCTTAGACAGTCTTTCGCCCAAGCACATCAGCCCGGACCACTCGCGTGGACTTCATACTCTCATATCCAACTATAGTTCTTAATCTCCCAAATtatatctccagcccagacctgtCCTCTGACCTGATTTAGCTGCTTATTTGACATGTCTGCTTGGATAGAGGCATCTTGAATTTAATGTGTCCAAAATTGGGATTCTTGAGTCTTCTCCACACACAAAACACAGCACTGAATAGGGCGTGGCAGACGTGGAGGGTGGTGGGTGCTCACCGATGGCCCAGGTTCCACCTCTCCTTGTAACCTCCACCCTCACTCTCCTGATACAGACTCGAAGTGTTTTCGTTCTTACGTGTAGTAAGCATTGCTAGAGCGCACCAGTACTGTgaactctccttcctctcctagacatcatatttatttatataagaggattttatttatccacttccTTGAAGGTAGACTTGGCTGTAGCTCTTACCTTGGCCACTGATATGTAAGTGAAAATTATACGTATTGCTTCTGGGTGGACATATTTAAGATTAGG |
| LNC 001899 | GTCCTCCGTCTATAAATACGCCACTGCGGGTTCTGAGGCAGGCACTGAGGCAGCGAGCGCAGAGACGACTTTGAGGCAGGAGGCGCAGCTTGAGGAGACGGCAGATAAGTTTCTCTATTGAAAAAAAGATAGGAATTCGTATAATTCTTTAGGAATTATAAATAGTGAAAGTACTGAGCTATTGCTGTGCATTAAGTTTTTAGAGTAGCTTTGATAGCTTAAGGAGATTTTATTTTAGGGGAACTATGAAGGCTTTCTTAAAGAGTagtttgaaaaagaagaggaaaatgataaTGTTAGAGCATGACGGAGGTTGAGATGAAGCATCTCCGTGGAGTAAATTTAAAAATCGCTAAGAGGGCTGCAGAGCCCGGAATTTAGAAACCATAAATGGAAGGGCAGCAGTTTAGATTAACGCTAAAACGTAGGGTGATATAAATAGAACTTAAACGTCATTTTAAAAAGTCGAGTTgtagatgataaaaatattttaacggccatcttttaaaaagatgatttaaatcGAAGATGATGCCTTCAGAATCATGGCGCAGTAAAAATTGTGTCATTTGAAGCCTGTTTGATCTTAAGGCTTTATTTGCTAAAATTGATGGAAATATTAACTAGCAATAGAGTAAATTTGGAGAATGAAGATTAGAAATTTGAAGAGGAAAACTCGGAAGACAGAAGTACAGGAAGGTGAAGAAAAGGCTTAGAGAAGAtaggaaaactggaagaaaaatcctccttttagaagacaaaaattaaacCAGAAGGTAGGAGGtagaagaaaaatcagacaaagcTAGAAAACTAGAAGATAGAAACAAGGTTGAAAATATGGTCAAGTGTTTcagatagaaaatgaaaaacaagcttATTGGCTACAAGATAGACGTCAAAATTAGAAGATAGACAAGTTCAAAGCCAAAAAATTGGATGAAGTACAAGAACAGAAAACCTAtggaaatttttataaagtattgtAGAAGCCCATCGATTTAAATATCTGGTGGTGCAGAAGTTAGATCTGAGAAGATGAGGGTGTTTACTGTAGACCAGCAACAACTTAGAAGAATATCCTGAAGCTAGGAGGGAAGTCAGTTTAAAATGGCGGCAAAAAGCTACTAAAAGGACTGAAGTAATTTAGAAACTAAAGTAGTTTTTGGAAGAGCTAAATCTGAAAGGCCTTAAATACAGTATCTtagtttcctttggaaaatttaAAGGACTTTATGACTGAAGTAATTGAAGAGCAAGAATAACCATCAACTTAACAATTTTGCATTGGACTTTGAGCTAAGAGGAGTTTTTAAATCCTGAGGACTAGGTGCTAATTAACAGCTGACCCAGGTGCTACACAGAAGTGGATTCAGTGAATCTAGGAAGACAGGAGCAGCGCAGGATTCCAGGAACCAGTGTTTGGTGAAACTAGGACTGAGGAGAAAGCATTTCGTGAAGATAGGAAAGAGGATCCCGGGAGCCAGTGCGATTTGGTGAAGGAAgctaggaagaaggaaggatCCCTAACGATTTGGAGGTGAAGCTAGGAGAGGATTCCAGGAAGGAGGAAGCCAGTGCGATGTGGTGATGAAGCTAGCGTGCGGCTTGGCGGCTTGGCAACCACGCGGAGGAGGCGAGCAGGTACCTCTCACTAAAGGCACCGAAGGCTTAAAGTAGGACAACCACGGAGCCTTCCTGTGGCAAGAGAGACAACAAAGCGCTATTATACTAAGGTCAAAGTGGTATCGGCCTTACAACCCTGTTCTCTGTTAGAAATGAGGACTTGCCTCAATACCCTTGGCAGACCGCGTTGAGGCTGTTTCTTTTGGGGAGTGAAGCATCCAGTAGAATGCTTGAGTAACTTGTATACCCCTGGGCTTGTCCTAACATTTAATGCAGCTGTTTTTATAGCAGCTCTTAGTAAAGCCCAAATCTTGAATGATGCttgaaggggagggaaagggggaaagcgGGCAACCACTTTTCCCTAGCTTTTCCAGAAGCCTGTTAAAAAGCAAGGTCTCCCCACAAGTGACTTCTCTGCCACATCGCCACCCCCTGCCTTTGGCCTAGCGCAGACCCTTCACCCCTCACCTCGATGCTGCCGGTAGCTTGGATCCTTGTGGGCATGATCCATATTGGTTTTAAGGTAACTGGTACTGAGCTCTTCTGGTGGGTTGCACTACTAGAAAAACCATTCATTTGCCTGCGTATGGTTAATGATATTAAGACCACACCTAAAAAGCTCTAAGATGCTTTAAATGTATGGCATTATTTATCCAGTGATTGAAACAAACcagtaaatgaagaaagaacatttaAGAATTGTAATGTTGGGTGGGAAGCTATAACTTACAAGCTGAAGAACATAGGCATTTTAACAGTTGAGGGCTTTTGGGTGGGATTGCAAAAATTCTCTGCTAAGACTTTTTCAGATGAACATAACAGACTTGGCCAAGCTAGCATCTTAGCTGAAGGAGATTCTCCAGTGCTCTTCAGTAGGGTTtataaaggtttttcttttcctgagaaaacaaaagatttgttttctcaggttttgctttttgcccttttcctagcttaaaaaaaaaaaaagcaaaagatgcTGGTGGTTGGCACTCCTGGTTTCCAGGACAGGGTTCAAATCCCTGTGGCATCTTTGCTTGACTTTAAGCTCTGCCTTCAGtactctttccattttcctcctcttttctttagATGCTAATCTGTAGACCTAGGGGAGTTGGAAGATAAAGTAGCTTCAAACTATACCTCTTCATTGAATttgtcagaagtctgaaatggctTGTTTTTGTCTTTACTGGGAACCTAATGTAACTGccttgtctttaattttcaggTATTCCCCATGCATCTAGTGTTTGGAGAAGTCTCactattaaatttattaaagttacatagtaataaaaattgaaaagctCTTCATTGGTGTGATTCCTTTGGGTGTAAACCAAATTTCTGTCCATGAGTAGACAGAAGATGGATGTTTTGTTGGGGGTTCTGTTGGGTTCCTGTCTTAAACTTGTGGTTTAATAACAAGTACACAGCACTGTCATGTAGCCTTATCTTCAAGTAGGGCAGAGACCTGGAGTAAGAAGTAGTGGGTGAGTGACCCAAGACTCAGTAACATCCATCCCCgtaaggaaaaaaaccccaaacacttCTCTAAGGAGCCTGCTTTCTGTTTAGCAATTGGatgtgggctgggctggagagaaAAATGTGGATGGGGAGGAGAGTTGTTAACCATTGCTCCTATGAAAGATTTAAAGCCAActagggggagagtatagctcaagtggtggacTGCAAGCatgttcagtccccagtacctcctgggCGGAAGGGAAGAACCGGCATTGTCTTTATCAGTTGTCAGTTCCCAAGCTACATGTTGGTCATGTGGTCACCTGTTCCGTGGAATGGCCTGCATACTAGTAGATAATCTCAGTTGCAGTTGAAATATCTTCACGGTCCCACCCATCCTCTACCTGGGGTGTCGTCTCTCAACCATATTCCCTGAATGGGTGGGAGAGGCTCATTCAAGTAAAGGTGGCCAGTGAGACCTAAGTGTGGGGCCCAGGTCTGGAGAAGCAGCACCCAGCATAGGTCTCCTAGCCGGGCCACACCCTCAGCccactggaggggaggggcctaAGCAAACAGCTCTTAGTATTACATTACTGAACTTGTAACTCGGGAAGGTGTTGCAAAAAGCAAGCTCTGATTTGGTCACTGCCAAGGCAACAGGGCTGGAATTCGAACTCTATTCTTGACCTCTTCAACTCTGTTGCTTTTACTTCAGTGCATGAATGTCCTCACATAAGAATCAACtatacatttcattcatttatatgaaacaaagacctatatagcacagggaactgtattcaatattttgtactctataatggaaaagaatctgaaagagtatatgtatatataaatatacatatgtatcatatcatcactttgctgtacacctgaaactaacacattataaatcaactatacttctattaaaaaaaaaaatcagctgttcACATAGGTCTGCAGCAGACCAGTCCCCAACACAGTGGTCCCTTGGGCTTGTTTAAGTGCCTGTTGTGAGGAGAAATCTCTGTTCTTCCAGCATTGGAAGACTATCTGGAAGCACAAAAGACTTTGCTGGAGAGCAAAGGAGTAAGAGGAAACTCTGGGGATTCATCAGGCGTTACATGATTCCTGGTGCTGAGCAGGCTCAAAATTAGCCAGGATTGGAAAATGTCAAGGCTGGACAGAGTCAGCGACAGCAGGGACCAGAGAAGTAGGCCAGAAGTCATTTCCTCCCCTGGAGCACAAAAATAATGGAGAAGGAGGCCAACATAAAATTCCTCTGAAGGTGTCCAAGTCAAGGGGCTAGgtcttgaaaatataaatgaacccCAAGCATGTCCTTTACATACTCCTCCAGCTGTATAAAGGGCTTTCACACCTTTTTTGCGGGGTGTGGGTAGGATTTCAGTGCTGCAATGGGTGGCTAAAATAAACCTGAATTCAAGTGATAGCTCCACCGTTTCCCAGCCTCTCCAAACTTGCCCCCAAgtctcccaggcccagggctaGTTGGTATGGACTTGGAAgtagatttctctctctctctctctc |
| LNC 000331 | GCAAAGAAGGCAGCAGAGGTTCCAGGGGGAAGGGAGCAGGCAATGTATGCAGCAGGTGACCCCCTGTTTTCTGAGCAGCATTTGGAAGATTTTCACGTGGCTTCCAGCCCTGGATCGTTCCCGGTGGGAAATTGACTGAGAGAAACCCTGCGGCCGAAGTGCAAAGACAAATGAAAGCGGGGCAGCGGCTGCTGAACGTCTAAAGGGCCCCTCCCAGCATGCCTTTGACTGCACGTGAGTTTTACTTTGCGGACTTTGGAGTTTAAGCTCCAGTTACGTATGACCGCGCTTGACTGGACGTCAGTAGGGGCGACGTCTCCCCAAGCGGTGCCAGGTTTGGGAAGTGTGTGGGAACATCTTAGGACGCCACTCCCCATCTCACTCTCCTCCCGCCTCCCGAGCCCCCACCCTTGGGGATGCAGAGCGTCCGGAGGTCTCTGATAAGGGCGGGCCACAGACTTCGGGTGTCAGCAGTGACCGGTTAGACGGCAGCAGCTGACTCACTGGGATCCCCACCACTAACCTGACTTTGCTGCAAAAAGCGCCCGGCGCCTCTCCGAGTGCCAGAGATGGAAGCCTTCGATCGCACCTTGTTGCAGGTGGTCCTCCCGAGAGGACCTCCTCCATCCAGCAGCCAGAGTGGAGGTCAGATCACGTTGCTGCTTTGCTCTGAAGCCTGCATCCTCCCCGCTCAAAAGACTCCTCAGCCCCACGACTGAAGCCAGATCCTCACTGGGCCTGCTGGGCTCACGTGACCTGACCCCACGGTGGTCCGACCTCATCTGTTGTCTCTGGATGTGATCTGCTCGCTACGTCCGCCTCTCCTGCCAACTGGCCTCCCTGCGGCTCCTGGACACGCACCTGTGCCCTGCACCTGTGGTCGGGGGCCCTGCAGCAGGGCTGAATGGCTTGTCCCATTGAAGTGTCACTCCGCAGCTAGGCCCCCAACCACTTTCATTAAATTTACACACCCAgcgtgccctccccaccctcttccctcttcccttctaatATCTGATGTAATGGACTTACTCTTTTGTGTGTTCCTCCTCCCCTGTTATGGGCTGGGTTGCACCCCCCCCTGAAATGGACATGTTGACGCCCCAGCCTCCACTGTGACTGGATCTGGAGCTGGGGCCTTTCAGGGGGCCATCGTGGTTAGATGAGGTCACAGGGTAGGGCCCTGATCCAACAGGCCGGTGGCcatagaagaagaggaagagacactggaTTTATCTGTCTCTCTGGGTGTCCAGAATAAGGCCACccagaggacacagtgagaagctaGCCGCCTGCAGGCCAGGGAGAGATGCTTCACCAGAAACTCACCCTGAGGACACCCTGGTCTtgcacttccagcctccagaactgtgagcagtaAGCGTGTGGCGTTCAAGCAGCCCGGGCTGCGCTCTTGTCACGGCAGCTGGTGCTGACCAGCGCACGCCCGTCACTCCACCGGAGTGTCAGCTCCGGGGAGACAGGAGTTTGGAGTTCCTGTCTCTCGTTTGAATAGCACCTACAAGGGCGCTTCcgtttTAAAGGTGAAAATTGCCAAGAGCTGCCAGTTACAGAGCAGGACCTCGGCCCCTGAAGGATGCTCTTCCAGACTGTCTGAGGAGGGACATCTCGCCCTggttcctctctcctccaggagAGGCTGGGTCCCCGCTGAAGCCTGCAGTCTGGCCAAGTCCTACTCCCTGCTTCACTTCTGAGTGGGAGGTTTGCTCCAGGTTTATTCTGACCCATAGGTAACATCACTGCTCCTCTAAGAACTTGAGGTCCGCCGGCTCTGGGGCTGGAACAAGCTAGAGCCCTGGCTGGAGGACGccagaaagagatggaaaatgactttctgggtgtgtgtgttgggggagggggaggggatggtggcAGAGGTGCGGGGCCCCTACTCGCAGGCTGGCCTTGAGCCTTGtccccccatctcccctcccttccgctcccctccccttccctccgcTCTCCTCGCCTCCTGTTCCAGCCTCTAGCTCTCCTGCTCGCCTTCCCCAGGACCACAGCCAGTCCACATGCTTTTCGTGGGTGTTAGACAAGCGCCCCCCTCTGGGCACACACAGCTCTGGTTGTAGCCTTTGGATCTGGGGCAGCTGGCTCCAGGAAGAGGCCAGAGCTCTGCAGGTGACACCAGGTAGGAAAAAATGGGGCGGGGGTCGTGTGACTGACGTTTGCGCAGGTGGAGGTCTGCGAGCTGCAGCGAGTCGTCCTGTTTTCAGTCTCCTCTGGGTCCAGGCTGAGAAACTGGAGGCACTGGCCTTGCTCTGGTGGCCTCGGAGCTGCCGCAGTCCCCTTGCTCGCCGGGAAATGCAGgcgtctgtgaaatggggtcaGGAGATGGAGGCGTAGTGGGCGGGCGGAACCGCCCCGCATACAATGAAACCCTTTCTTCCCGCAACACAAC |
