# Supplementary material for: Key genes differential expressions and pathway involved in salt and water-deprivation stresses for renal cortex in camel
Source: BMC Mol Biol. 2019 Apr 8;20:11. doi: 10.1186/s12867-019-0129-8 (PMC6454748; doi:10.1186/s12867-019-0129-8)
Supplement: Supplementary file 2 — Additional file 2: Figure S1. Normalized mean plasma Na+ concentration of camel under salt stress, water-deprivation stress and free diet. [file 12867_2019_129_MOESM2_ESM.docx]

Figure S1. Normalized mean plasma Na+ concentration of camel under salt stress, water-deprivation stress and free diet.
